# A Comparative Analysis Between Ultrasound and Electromyographic and Nerve Conduction Studies in Diagnosing Carpal Tunnel Syndrome (CTS): A Systematic Review and Meta-Analysis

**DOI:** 10.7759/cureus.30476

**Published:** 2022-10-19

**Authors:** Hany A Zaki, Eman Shaban, Waleed Salem, Farah Bilal, Mohamed Fayed, Mohamed Hendy, Mohammed Gafar Abdelrahim, Maarij Masood, Yousra Mohamed khair, Nabil A Shallik

**Affiliations:** 1 Emergency Medicine, Hamad Medical Corporation, Doha, QAT; 2 Cardiology, Al Jufairi Diagnostic and Treatment, Doha, QAT; 3 Emergency, Hamad Medical Corporation, Doha, QAT; 4 Anesthesia and Critical Care, Saint George Hospital, Balamand University, Beirut, LBN; 5 Accident and Emergency, Hamad Medical Corporation, Doha, QAT; 6 Medical Education and Simulation, Weill Cornell Medicine - Qatar, Doha, QAT; 7 Anaesthesia, Hamad Medical Corporation, Doha, QAT

**Keywords:** carpal tunnel syndrome, systematic review and meta-analysis, electrodiagnostic test, electromyography and electro-stimulation, nerve conduction studies (ncs), high-resolution sonography

## Abstract

Carpal tunnel syndrome (CTS) is the most common upper extremity neuropathy. The disease initially manifests as a sensory disorder in the form of paresthesia, numbness, or tingling of the fingers. The diagnosis is usually made based on history and clinical symptoms, which are confirmed using nerve conduction studies (NCS) and electromyography. More recently, ultrasound has gained more use in CTS diagnosis due to its advantages, which include patients’ comfort during diagnosis, better visualization of anatomy and nerve forms directly, and cost-effectiveness. However, a literature review shows that the diagnostic accuracy of ultrasound over NCS is still in question; therefore, the present systematic review was carried out to compare the diagnostic accuracy of ultrasound to NCS and electromyography.

A systematic literature search was performed on five electronic databases: PubMed, Medline, Web of Science, Embase, and Google Scholar. The search strategy limited the retrieval of literature published between 2000 and 2022. Of the 1098 articles retrieved from the electronic databases, only 12 met the inclusion criteria. A meta-analysis of outcomes from the included studies showed that the pooled sensitivity and specificity of the ultrasound were 0.80 (95% CI: 0.73, 0.88) and 0.90 (0.83, 0.96), respectively. On the other hand, combing the outcomes of electromyography and NCS resulted in sensitivity and specificity values of 0.89 (95% CI: 0.84, 0.95) and 0.77 (95% CI; 0.64, 0.90), respectively.

The results show that ultrasound has comparable sensitivity and slightly higher specificity than NCS and electromyography; therefore, ultrasound can be used as an alternative diagnostic test for CTS. However, it cannot replace NCS and electromyography since more research needs to be done on doubtful and secondary cases of CTS.

## Introduction and background

Carpal tunnel syndrome (CTS) is the most common upper extremity neuropathy [1]. The initial symptoms of this disease include sensory disorder, which occurs in the form of paresthesia, numbness, or tingling of the fingers [2]. In more severe conditions, motor symptoms are observed and can lead to atrophy of the thenar muscle or other muscles innervated by the median nerve. Extreme conditions can also negatively impact the patient’s quality of life. The prevalence of this disease has been estimated to range from 5% to 15%, depending on the diagnostic criteria utilized [3,4]. A more recent study reported that in the United States (US) alone, one to three cases of CTS are observed for every 1000 people yearly [5]. However, it is recorded that the incidence of CTS can be as high as 150 cases per 1000 people yearly. Previous research has also documented that CTS is more dominant in women than men occurring at a ratio of 5:1 [6,7]. However, other studies show that the ratio of women to men with CTS is 3:10 [5]. Additionally, evidence indicates that CTS is more prevalent in people aged above 40 years [6] and can be associated with manual activities. Other factors that increase the risk of CTS are underlying conditions such as rheumatological and endocrinological diseases, inflammatory alterations, bone, muscle, neurovascular abnormalities, tumoral lesions, and pregnancy.

The diagnosis of CTS is usually based on history and clinical symptoms, which are confirmed by nerve conduction studies (NCS) and electromyography. However, the diagnosis of CTS primarily depends on the results obtained from the NCS. Electromyography is mainly utilized to exclude other conditions such as polyneuropathy, plexopathy, and radiculopathy. Even though NCS is vital in diagnosing CTS, it has shown a specificity of 95% and a sensitivity ranging from 49% to 86% [8]. NCS has also shown substantial false negative rates of 10-20% and false positives in some cases [9-11]. In addition, NCS results have the disadvantage of not providing spatial information about the nerve or its surroundings despite indicating the level of lesions.

In recent years, imaging techniques, such as magnetic resonance imaging (MRI) and ultrasonography, have gained importance in the diagnosis of CTS. The increasing use of ultrasound in diagnosing CTS can be attributed to several factors. First, ultrasound allows better visualization of anatomy and nerve forms directly. Second, ultrasound is more comfortable for patients, as it only involves a transducer that comes into contact with the skin of the patient’s hand. Previous studies have also reported that ultrasound has higher sensitivity and specificity than NCS and electromyography. For instance, Fowler et al. reported sensitivity and specificity values of 77.6 and 86.8%, respectively [12]. However, disadvantages in the diagnostic accuracy of ultrasound over NCS have also been documented. A previous review reported that ultrasound had less sensitivity and specificity than NCS [13].

The review of past literature has shown that the diagnostic accuracy of ultrasound over NCS is still in question; therefore, the present systematic review will compare the diagnostic accuracy of ultrasound to NCS and electromyography in CTS. For this review, NCS and electromyography will be grouped as electrodiagnostic tests, after which the most reliable diagnostic test will be determined by conducting a meta-analysis on the specificity and sensitivity.

## Review

Methodology

Literature Search

A detailed search for relevant and original articles adhered to the Preferred Reporting Items for Systematic Reviews and Meta-Analyses (PRISMA) guidelines and was done by utilizing two methods. The first method involved a database search on PubMed, Medline, Web of Science, Embase, and Google Scholar. During the database search, the Boolean expressions “AND” and “OR” were used to combine specific keywords, thus forming a detailed search strategy. The search strategy was as follows: (carpal tunnel OR carpal tunnel syndrome OR CTS) AND (Ultrasound OR ultrasonography OR sonography OR High-resolution sonography) AND (nerve conduction studies OR nerve conduction velocity) AND (electromyography OR electroneuromyography OR electrophysiological test OR electrodiagnostic test). All the search results were limited to studies published between 2000 and 2022. The second method involved reviewing relevant articles’ reference lists for additional studies. Additionally, we did not retrieve any grey or unpublished literature, as we wanted to have rigorous scientific research.

Eligibility Criteria

Two reviewers analyzed articles relevant to the topic using the inclusion and exclusion criteria. Studies were eligible for inclusion if they satisfied the following criteria: Articles written and published in English. This criterion was essential, as it helped us avoid the direct translation of scientific terms, which could lead to a loss of meaning and context. Studies comparing ultrasound to electromyography, nerve conduction studies, or a combination of both (electrodiagnostic tests) in diagnosing CTS. Studies with adequate sample sizes (more than 10). This specification was made to enhance the statistical power of our meta-analysis. Studies where the specificity and sensitivity of the diagnostic tests were well outlined.

On the other hand, studies could not be included for the following reasons: Studies published in languages other than English. Studies whose diagnostic outcomes did not include specificity and sensitivity values. Studies that individually evaluated either ultrasound, electromyography, or nerve conduction studies. Articles whose design was either systematic review and meta-analysis, case reports, and letters to the editor. Abstracts without evidence of full articles.

Data Extraction

Two reviewers were tasked with independently retrieving relevant data from articles that met the eligibility criteria. The primary data collected included: Study ID (first author’s name and year of publication), characteristics of patients (age, sex, and sample size), the diagnostic classification system used, ultrasound measures of diagnosis, NCS or electromyography measures of diagnosis, and main outcomes. The main results of this systematic review were the specificity and sensitivity values. Any conflicting data arising during the data extraction process was reconciled by consulting a third reviewer.

Quality Assessment

All studies included for review in the present study were non-randomized; therefore, a methodological quality assessment of each study was performed using the Newcastle-Ottawa Scale [14]. The assessment criterion was based on eight evaluation questions categorized into selection, comparability, and outcomes. Studies were awarded 1 point for each criterion fully answered and 0 for a criterion not answered. A score of ≥ 7 meant that the study had high methodological quality while a score of between 4 and 6 meant that the study was of moderate methodological quality. On the other hand, studies with a score of ≤3 were considered low quality [14].

Data Analysis

The pooled effect of all outcomes in the present study was done using STATA software (version 16.0; StataCorp LP, College Station, TX). The weighted random effect model was used to calculate the pooled specificity and sensitivity values from the studies included in the review. The weight applied during the analysis depended on the sample sizes recorded in each study. A random effect model was specifically chosen for the present meta-analysis to accommodate the expected heterogeneity, which was measured using the I2 statistics [15]. Heterogeneity values of less than 50%, 51-70%, and above 70% were considered low, moderate, and high, respectively [15]. A confidence interval of 95% was also chosen to improve the statistical power of the present meta-analysis. The results of the meta-analyses were presented in forest plots. Significance was also measured using a p-value of which a significant difference was defined by p < 0.05.

Results

Search Results

**Figure 1:**
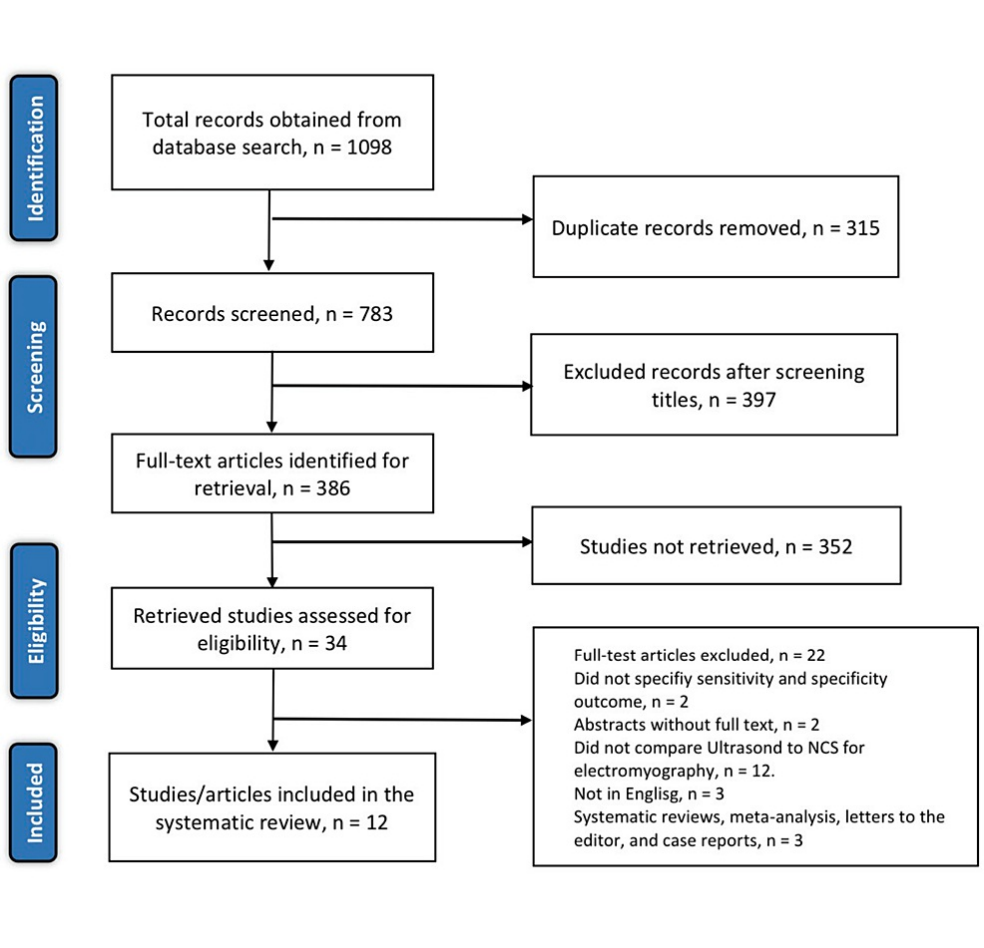
PRISMA flow diagram of the literature search results PRISMA: Preferred Reporting Items for Systematic Reviews and Meta-Analyses

The initial search through the aforementioned databases yielded 1098 relevant articles. The two reviewers tasked with the literature search then screened for duplicates and excluded 315 articles. The titles and abstracts of the remaining articles were screened, and 397 articles were eliminated. Of the remaining 386 articles, 352 were not retrieved and the other 34 articles were assessed using the eligibility criteria outlined earlier. The assessment using the eligibility criteria yielded only 12 articles for inclusion. The other 22 articles were excluded on the following basis: three were non-English articles, two were abstracts without evidence of full article, three were either systematic reviews, letters to the editor, or case reports, two did not specify the specificity and sensitivity values, and 12 studies did not compare ultrasound to NCS or electromyography. See Figure 1 shows the study selection process following the PRISMA guidelines.

Table 1 shows the study characters.

**Table 1:** Study characters NR: not reported; CSA; cross-sectional areas; FR; flattening ratio; DML: distal motor latency; DSL: distal sensory latency; SCV: sensory conduction velocity; SNAP: sensory nerve action potentials; CMAP: compound muscle action potential; NCS: nerve conduction studies; NCV: nerve conduction velocity; EDX: electrodiagnostic tests

Author ID	Study design	Subject characteristics (subjects/wrists)	Classification of diagnosis (n)	US measures (cut-off)	NCS or electromyography measures	Main outcomes
Kwon et al., 2008 [8]	A prospective, case-controlled study	29/41 (4 males and 25 females, 53 (25–75) years)	NR	CSA (10.7 mm^2^)	Sensory amplitude. DML, DSL	Using a CSA cut-off point of 10.7mm2, the sensitivity and specificity of sonography and NCS were 66% and 63%, respectively. A combination of NCS and sonography gave sensitivity and specificity values of 90% and 62%, respectively.
El-Shintenawy et al., 2019 [14]	Cohort study	40/56 (39 female and 1 male; 36.02 ± 8.4 years).	Negative (2), Minimal (2), Mild (28), Moderate (16), Severe (8)	CSA (>9 mm^2^) FR1 (>3) FR2 (>4)	DML, DSL, Sensory amplitude, SCV	The sensitivities for ultrasound parameters were (80.4%, 50%, and 91.3%, p<0.001 for CSA, FR1, and FR2, respectively). All the ultrasound parameters showed a specificity of 100%. The sensitivities for NCS parameters were 53.6, 55.4, 73.2, and 94.6% for DML, DSL, sensory amplitude, and SCV, respectively. The specificity was significantly higher in DML and SCV (100%, p<0.001) while it was significantly lower in sensory latency (63.3%, p=0.04).
Swen et al., 2001 [15]	Cohort study	63 (44 women and 19 men; 52 ± 13 years)	NR	CSA (>10 mm^2^)	DSL, DML, SCV, MCV	NCS showed a significantly higher sensitivity than sonography (0.98 vs. 0.70, respectively). Sonography recorded a higher specificity than NCS (0.63 vs. 0.19).
El Miedany et al., 2004 [10]	Cross-sectional case-control study.	78/96 (51 female and 27 males; 44.9 ± 6.16 years)	Negative (6), Mild (30), Moderate (33), Severe (27)	CSA (>10.03 mm^2^)	DSL, DML, SCV	6 hands showed negative results on the electrophysiological tests while only 2 hands showed negative results upon Ultrasound assessment. Ultrasound assessment on patients with moderate and severe diagnoses resulted in a 96.6% sensitivity and 99% specificity.
Pimentel et al., 2018 [16]	Prospective clinical trial	115 females (40 – 79 years)	NR	CSA (≥ 10 mm^2^)	SCV, DML	NCS showed higher sensitivity and specificity than ultrasound (92.3% and 90.9% vs. 84.6% and 81.8%, respectively)
Visser et al., 2007 [17]	Prospective cohort study	168 (39 male and 129 females; 52 +- 14 years)	Normal (94), Mild (53), Moderate (8), Severe (12)	CSA (>0.1 cm^2^)	SNAP, DSL, DML, Distal CMAP, median nerve	16 of 28 patients with negative EMG had a positive sonogram. The sensitivity and specificity for sonography were 78% and 91%, while EMG tests for DML median nerve > 3.8 msec showed a sensitivity and specificity of 74% and 97%, respectively. 1 patient had a slight preference for EMG while 5 patients had a very strong preference for sonography.
Filho et al., 2014 [6]	Cross-sectional study	56/70 (2 males and 54 females)	NR	NR	NR	EMG had higher sensitivity than ultrasound and physical examination tests (98.6% vs. 67.1% vs. 95.7%, respectively).
El Badry et al., 2016 [18]	Prospective study	100 (24 men and 76 women; mean age 41.3 years)	Normal, Mild, Moderate, Severe	NR	SCV, DML	NCV showed clinically 90 positive and 10 negative CTS cases while ultrasound showed 86 positive and 14 negative cases. The sensitivity and specificity of NCV and ultrasound were 90% and 79.2% vs. 86% and 77.4%, respectively.
Kele et al., 2003 [19]	Comparative study	77/110 (59 women and 18 men; 52 (22 – 84) years).	NR	CSA (≥0.11 cm^2^)	SCV, DML	A higher predictive value of ultrasound was observed for CSA > 0.11 cm2 (89.1% and 98%, sensitivity and specificity, respectively. The electrophysiological test showed a sensitivity of 90.0%.
Azami et al., 2014 [20]	Prospective cross-sectional study	90/120 (83 women and 7 men; 56.8 + 10.6 years)	Mild (57), Moderate (39), Severe (34)	CSA (9.15 and 8.15 mm^2^), FR (1.02, 1.01 and 0.94)	SCV, DML	CSA at the tunnel inlet with a threshold of 9.15 mm2 provided the best diagnostic accuracy with a sensitivity of 99.2% and specificity of 83.3%. The sensitivity and specificity of the ultrasonography at a 1.02 cut-off for the FR at the proximal were 98.3% and 46.7%, respectively.
Fowler et al., 2014 [21]	Comparative cohort study	85 (31 men and 54 women; 56 (18 – 86) years)	NR	CSA (≥10 mm^2^)	DSL, DML	Using the EDX tests as the reference standard, ultrasound had a sensitivity of 85% and specificity of 83%. Electrodiagnostic tests showed sensitivity and specificity of 89% and 80%, respectively.
Moran et al., 2009 [22]	Prospective study	46/70 (40 women and 6 men; 45 (30 – 80) years)	Negative (20), Mild (15), Moderate (13), Severe (22)	CSA (9.8, 12.30, 11, and 13 mm^2^)	DML	NCS confirmed CTS in 50 of 70 hands. The sensitivity and specificity for CSA >9.8, ≥12.30, >11 and >13 was 92% and 45% vs. 62.0% and 95% vs. 86% and 40% vs. 60% and 90.0%, respectively.

Quality Assessment Results

The quality assessment showed that three studies were of high quality while the other nine studies were of moderate quality. The assessment also showed that none of the studies had a low methodological quality assessment (Table 2).

**Table 2:** Methodological quality using the Newcastle Ottawa Scale for cohort studies

Author ID	Selection (Maximum 4)	Compatibility (Maximum 1)	Outcome (Maximum 3)	Total Score	Quality
Kwon et al., 2008 [8]	3	1	2	6	Moderate
El-Shintenawy et al., 2019 [14]	2	1	3	6	Moderate
Swen et al., 2001 [15]	3	1	3	7	High
El Miedany et al., 2004 [10]	3	1	2	6	Moderate
Pimentel et al., 2018 [16]	3	1	3	7	High
Visser et al., 2007 [17]	2	1	2	5	Moderate
Filho et al., 2014 [6]	3	1	2	6	Moderate
El Badry et al., 2016 [18]	4	1	2	7	High
Kele et al., 2003 [19]	3	1	2	6	Moderate
Azami et al., 2014 [20]	2	1	3	6	Moderate
Fowler et al. 2014 [21]	2	1	2	5	Moderate
Moran et al., 2009 [22]	3	1	2	6	High

Ultrasound

**Figure 2:**
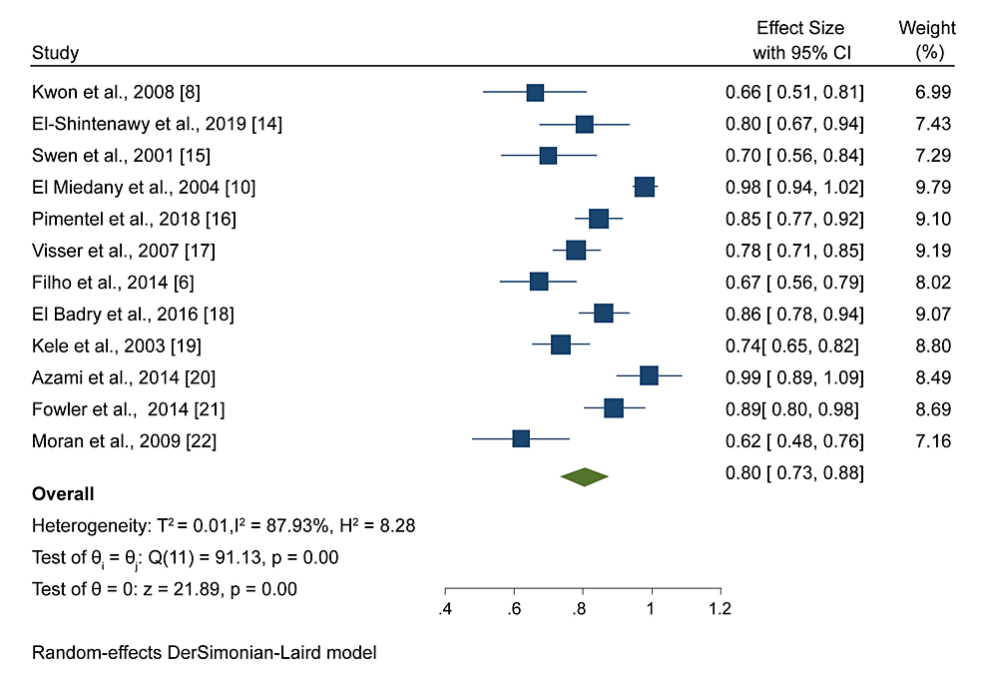
A forest plot showing the pooled ultrasound sensitivity Kwon et al., 2008 [8], El-Shintenawy et al., 2019 [14], Swen et al., 2004 [15], El Miedany et al., 2004 [10], Pimentel et al., 2018 [16], Visser et al., 2007 [17], Filho et al., 2014 [6], El Badry et al., 2016 [18], Kele et al., 2003 [19], Azami et al., 2014 [20], Fowler et al., 2014 [21]. Moran et al. 2009 [22]

All 12 included studies evaluating 947 patients suspected to have CTS used ultrasound as the diagnostic test. A meta-analysis of results from these studies showed that the pooled sensitivity was 0.80 (95% CI: 0.73, 0.88) (Figure 2).

**Figure 3:**
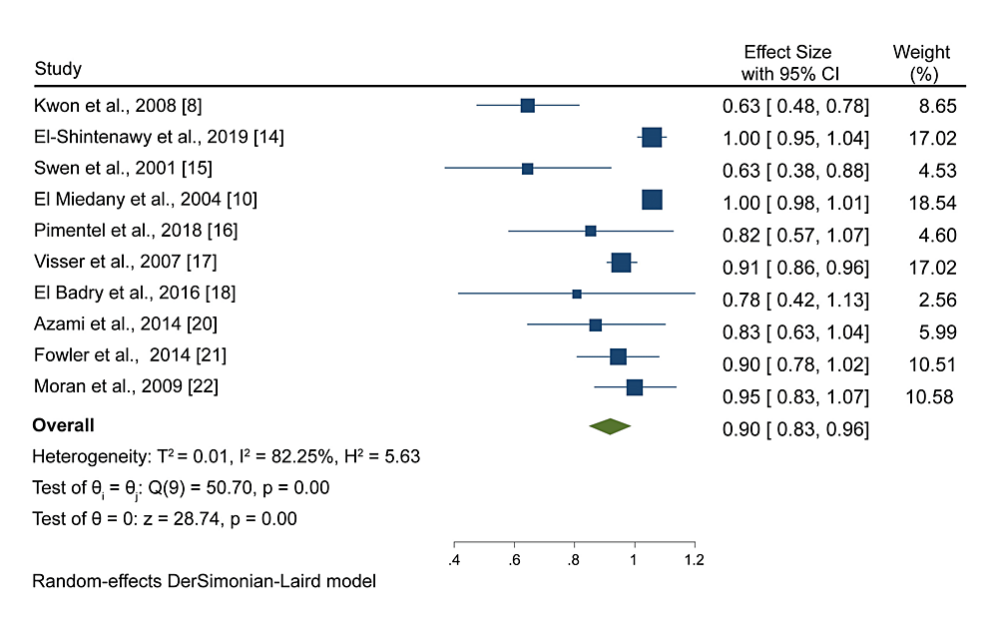
A forest plot showing the pooled ultrasound specificity Kwon et al., 2008 [8], El-Shintenawy et al., 2019 [14], Swen et al., 2004 [15], El Miedany et al., 2004 [10], Pimentel et al., 2018 [16], Visser et al., 2007 [17], El Badry et al., 2016 [18], Azami et al., 2014 [20], Fowler et al., 2014 [21]. Moran et al. 2009 [22]

On the other hand, ultrasound reported a pooled specificity of 0.90 (0.83, 0.96) after analyzing outcomes from 10 studies (Figure 3).

NCS and Electromyography

**Figure 4:**
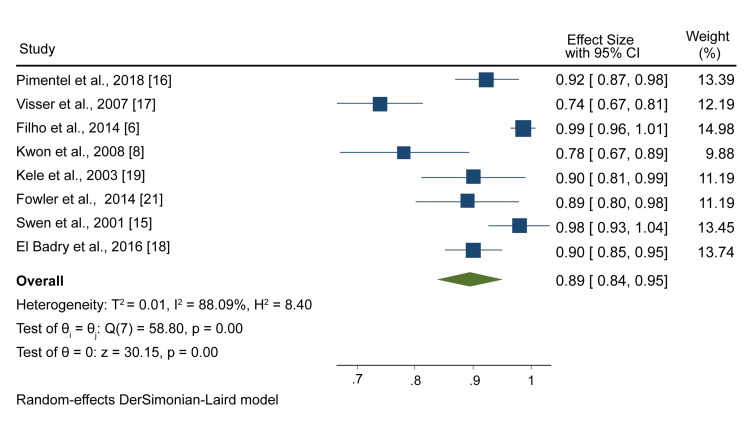
A forest plot showing NCS and electromyography pooled sensitivity Pimentel et al., 2018 [16], Visser et al., 2007 [17], Filho et al., 2014 [6], Kwon et al., 2008 [8], Kele et al., 2003 [19], Fowler et al., 2014 [21]. Swen et al., 2001 [15], El Badry et al., [18] NCS: nerve conduction studies

The sensitivity of either NCS or electromyography or a combination of both was reported in eight studies. A meta-analysis of outcomes from these studies showed that the pooled sensitivity was 0.89 (95% CI: 0.84, 0.95) (Figure 4).

**Figure 5:**
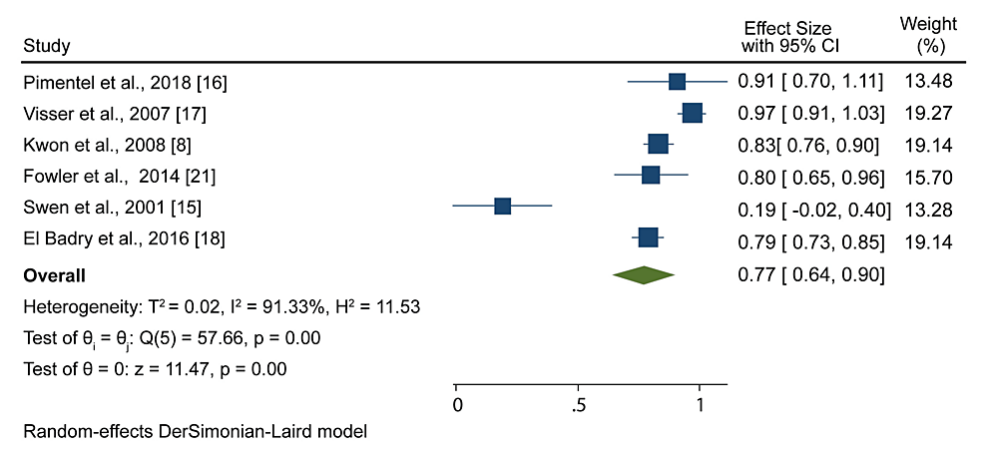
A forest plot showing NCS and electromyography pooled specificity Pimentel et al., 2018 [18], Visser et al., 2007 [17], Kwon et al., 2008 [8], Fowler et al., 2014 [21], Swen et al., [15], El Badry et al., 2016 [18] NCS: nerve conduction studies

On the other hand, the specificity of NCS and electromyography was reported in six studies, and the meta-analysis resulted in a pooled specificity of 0.77 (95% CI; 0.64, 0.90) (Figure 5).

An accurate diagnosis of CTS is vital, especially if a patient is a candidate for surgery. Currently, ultrasound has gained increased use in diagnosing CTS. Many authors believe that ultrasound can be used as an alternative to NCS and electromyography for the primary evaluation of CTS in daily practice. The present meta-analysis has revealed that ultrasound has a comparably similar sensitivity to NCS and electromyography. However, the results show that ultrasound has slightly higher specificity than NCS and electromyography.

The high ultrasound sensitivity and specificity reported in the current study are supported by a previous meta-analysis that reported that the composite pooled sensitivity and specificity of ultrasound in the diagnosis of CTS was 77.6% (95% CI 71.6%-83.6%) and 86.8% (95% CI 78.9%-94.8%), respectively [12]. Similarly, a meta-analysis that used a cut-off cross-sectional area (CSA) of 9.5 mm^2^ to 10 mm^2^ as the inclusion criteria reported that ultrasound had a sensitivity of 0.84 (95% CI; 0.81, 0.87) [23]. However, the specificity recorded in the study was lower than that reported in the present study (78.4 (68.4 - 88.3) vs. 90% (83% - 96%)). The difference in that study can be attributed to the strict inclusion criteria that may have influenced its statistical power. Even though the present study has shown high specificity and sensitivity, various studies have shown varied results depending on the ultrasonographic measures used. The reported ultrasonographic measures used in CTS diagnosis include CSA of the nerve at various levels of the carpal canal, flattening ratio (FR), swelling ratio, and increased palmar bowing of the flexor retinaculum.

Unlike other measures, CSA of the median nerve is the most commonly used due to its ease of measurement and is most consistent and notable in both patients and controls. However, various studies have used different cut-off CSA when diagnosing CTS. For instance, Azami et al. [20] reported that the best ultrasound diagnostic accuracy was recorded when using the CSA at the tunnel inlet with a threshold of 9.15 mm^2^ (99.2% and 88.3%, sensitivity and specificity, respectively). Previous research work by Duncan et al. also seemed to agree with these results by reporting that a cut-off point of 9.0 mm^2^ was the most predictive criterion for CTS (82.4% and 97%, sensitivity and specificity, respectively) [24]. On the other hand, Kwon et al. reported that the CSA at the carpal tunnel inlet with a 10.7 mm^2^ threshold showed the best diagnostic accuracy (66% and 63%, sensitivity and specificity, respectively) [8]. A previous study by Nakamichi and Tachibana using a cut-off value of 13 mm^2^ also reported a sensitivity of 57% and specificity of 97% [25]. The high specificity recorded in the study showed that ultrasound had the ability to diagnose CTS. Additionally, Moran et al. reported the sensitivity and specificity of ultrasound at CSA cut-off values of 9.8, 12.30, 11, and 13 mm^2^ [22]. The results showed that the highest and lowest sensitivities were recorded using a cut-off of 9.8 mm^2^ and 13 mm^2^ (92% vs. 60%, respectively. However, the cut-off value of 9.8 mm^2^ showed a low specificity (45%) while the 12.30 mm^2^ threshold showed the highest specificity (95%). Mondelli et al. also demonstrated that a cut-off value of 8.5 mm^2^ gave the best prediction for CTS, yielding pooled sensitivity and specificity values of 97-100% and 98-100%, respectively [26].

Even though CSA of the median nerve is the widely used ultrasonography measurement, evidence from other studies has shown that FR could be significant in diagnosing CTS. Azami et al. reported that using FR at the proximal to the carpal tunnel with a cut-off point of 1.02 yielded ultrasonographic sensitivity and specificity of 98.3% and 46.7%, respectively [20]. Adjusting the cut-off points to 1.01 and 0.94 yielded sensitivities of 94.2% and 99.2% and specificities of 55% and 75%. Comparing these results to those obtained when using the CSA proximal to the carpal tunnel, it is safe to say that FR also offers better diagnostic criteria for CTS. A more recent study by El-Shintenawy and colleagues also reported that using FR2 at the hamate level, as the diagnostic criterion had a better sensitivity and accuracy than CSA (91.3% and 95.5% vs. 80.4% and 87.2%, respectively) [14]. However, the study explained that CSA is the main sonographic measurement used in the diagnosis of CTS. There has also been evidence that the thickness of the flexor retinaculum has the ability to diagnose CTS. According to Keleş et al., flexor retinaculum thickness with a threshold value of 3.7 mm was considered significant in CTS diagnosis (71.4% and 55%, sensitivity and specificity, respectively) [27]. Additionally, Azami et al. reported the use of flexor retinaculum thickness in the diagnosis of CTS [20]. Results of the study showed that a cut-off point of 1.26 mm in the thickness of the flexor retinaculum gave an ultrasonographic sensitivity and specificity of 99.2% and 66.7%, respectively.

The meta-analysis in the present study also shows that NCS and electromyography have high sensitivity and specificity. These results can be supported by a previous meta-analysis that showed that electrodiagnostic tests had sensitivity and specificity of 80.2% (95% CI 71.3%-89.0%) and 78.7% (95% CI 66.4%-91.1%), respectively [12]. Previous studies also seem to agree with the results of the present study by reporting that NCS sensitivity ranges from 56% to 85% [14]. However, the reported NCS specificity ranged from 94% to 99% thus, contradicting our results. The contradiction can be attributed to the fact that the present study combined the outcomes of NCS and electromyography. Similar to ultrasound, NCS and electromyography use various criteria for the diagnosis of CTS. The NCS measurement criteria include sensory conduction velocity (SCV), distal motor latency (DML), sensory amplitude, and distal sensory latency (DSL). El-Shintenawy et al. reported that using SCV with a cut-off of ≤50 as the diagnostic criterion, high sensitivity and accuracy were observed (94.6% and 89.5%, respectively) [14]. The other electrophysiological parameters, such as DML (>4.2), DSL (>2.9), and sensory amplitude (≤15), showed that the sensitivity and accuracy were 53.6% and 100%, 55.4% and 63.3%, and 73.2% and 100%, respectively. Similarly, Visser et al. recorded that using DML with a cut-off value of 3.8 msec showed high sensitivity and specificity of 74% and 97%, respectively [17].

The effect of combining NCS or electromyography with ultrasound on the diagnostic accuracy of CTS has also been evaluated. Kwon et al. [8] hypothesized that combining ultrasound would improve specificity with or without improvement in sensitivity; however, the results showed that NCS failed to improve sensitivity or specificity compared with NCS alone. The inclusive combination (either of the tests is positive) showed that the specificity significantly decreased without any significant change in the sensitivity. On the other hand, the exclusive combination (both of the tests are positive) showed that specificity decreased, but there was no significant change in the sensitivity. These results indicated that there was no benefit of adding NCS to ultrasound in diagnosing CTS. Visser et al. also reported that it is not beneficial to combine ultrasound with electrodiagnostic tests [17]. Even though the results showed that combining ultrasound with electrodiagnostic tests would improve the kappa index, the improvement was not significant; thus, it is not advisable to combine the diagnostic tests, especially in patients with atypical CTS.

Comparing the results of the present meta-analyses, it is safe to say that ultrasound can be used as an alternative diagnostic test for patients with CTS since the results are comparable. To support this finding, there has been evidence that ultrasound is preferred by patients over NCS and electromyography. Visser and colleagues carried out a random investigation among 20 patients on their preferred diagnostic test [17]. They found that of nine patients that had undergone electrodiagnostic tests, two had a slight preference for electrodiagnostic testing while seven claimed that they did not care about the test. On the other hand, of 11 patients questioned about ultrasound, two responded that they had a slight preference, five strongly preferred sonography, and the other 5 had a very strong sonography preference. Ultrasound is preferred in most cases due to its numerous advantages. First, ultrasound is less costly than NCS and electromyography. A recent study reported that ultrasound is cost-effective when the hand surgeon uses it as the first-line diagnostic test. It is also important to note that during the cost-effectiveness analysis, it was assumed that patients had been referred to the radiologist for an ultrasound examination; therefore, the formal charges were incurred in the testing process. According to the results of that study, the average cost of conducting an ultrasound for CTS diagnosis was $476.30 (256-1275) [28]. Second, it has been reported that ultrasound is faster than NCS and electromyography in diagnosing CTS. Fowler et al. reported that a learning curve for about 20 patients revealed that ultrasound was able to perform CTS examinations in less than 90 seconds for every patient [21]. The study also suggested that decreasing the number of examinations from three to one could reduce the diagnosis time to 30 seconds for every patient. On the other hand, electrodiagnostic tests are estimated to take about 30 minutes even when they are performed by well-trained technicians. Ultrasound also provides a more painless alternative and a teaching moment between the physician and the patient, as it allows the patient to visualize the relationship between the nerves and tendons in the carpal tunnel. On the other hand, electrodiagnostic tests, such as electromyography, are painful and cause patient discomfort. However, the discomforts that arise in electrodiagnostic testing are usually overlooked. In other studies, ultrasound has been recommended as the first diagnostic procedure, especially for patients with typical CTS. As discussed earlier, there is a relationship between an increase in the CSA of the median nerve with CTS; therefore, CSA of the median nerve is essential. However, for patients with atypical symptoms involving diagnoses, such as C6/C7 radiculopathy, underlying polyneuropathy, sensory neuropathy, or proximal lesions of the median nerve, electrodiagnostic testing can be used for patients with typical CTS [17].

Additionally, the high rates of false negatives observed in electrodiagnostic tests have led to the preference for ultrasound in CTS diagnosis. Filho et al. reported that the rate of false negatives observed in electromyography ranged from 0%-4.3% [6]. However, these rates were significantly lower than those reported in previous literature. Werner and Andary reported that electrodiagnostic testing recorded as high as 10-15% false negatives in CTS diagnosis [29]. These high rates were attributed to the fact that the study included patients with intermittent symptoms in whom axonal lesions do not occur. Studies by Dhong et al. and Padua et al. also reported cases of false negatives and recommended that electrophysiological examinations should be used as reference tests and the diagnosis should be made based on the typical symptoms [30,31]. However, this does not mean that ultrasound is not subject to false negatives. As a matter of fact, some studies have shown that ultrasound can have higher false negatives than NCS. Swen et al. reported that 14 false negatives were observed in the sonography group while only one false negative was recorded for NCS [15]. This high false-negative was attributed to inaccurate measurements and the presence of patients with normal sizes of the mean CSA of the median nerve.

Ultrasound also has limitations that may lead to a preference for NCS and electromyography. One limitation of ultrasound is in CTS management. Unlike NCS, ultrasound is unable to distinguish between CTS-mimicking diseases. It is due to this reason that Kwon and colleagues suggested that sonography can neither be used as a complementary nor alternative diagnostic test to NCS. Another major limitation is that ultrasound, being an observer-dependent examination, can result in biased and conflicting outcomes and opinions. Researchers have claimed that information bias may arise if the ultrasound images are evaluated by different professionals utilizing different equipment [6]. Additionally, ultrasound lacks the ability to be used in the grading of CTS severity. No literature has been able to show that CSA of the median nerve, which is a parameter used in ultrasound examinations, correlates with CTS or nerve recovery after carpal tunnel release. However, many studies have shown that NCS parameters, such as DSL, DML, sensory amplitude, and SCV have been associated with the severity of CTS. According to a study by Kollu et al. [32], Mild CTS was associated with median DML <4.5 and sensory nerve conduction velocity (NCV) <40, Moderate severe CTS was associated with median DML >4.5 and < 6.5 with preserved sensory nerve action potentials (SNAP), severe CTS was associated with DML >4.5 and <6.5 with an absent SNAP, very serve CTS was associated with DML > 6.5 with compound muscle action potential (CMAP) > 2.0 mV and extremely severe CTS was associated with median CMAP from APB <0.2 mV.

Limitations

The present systematic review and meta-analysis were subject to several limitations. First, the eligibility criteria of the current study specified that only articles published and written in English were to be included for review. This criterion may have led to the exclusion of relevant articles that would have improved our meta-analysis’s scientific research and statistical power. The meta-analyses also showed high heterogeneity, which can be attributed to the fact that the measures used to diagnose CTS varied from study to study. Our study also did not specify the cut-off CSA of the median nerve to be used in identifying the sensitivity and specificity of ultrasound, thus contributing to the high heterogeneity. The other limitation is that most of the studies included in the present research were of prospective and cross-sectional design, thus introducing the publication bias that comes with such studies. However, the quality assessment shows that most studies were of high quality, meaning they carried a low-risk bias. The sensitivity and specificity results recorded in our study should also be interpreted with extreme care due to the low number of studies used in the meta-analysis. The low number of included studies due to strict inclusion criteria may have influenced the statistical power of our results. The current study also did not carry out a subgroup analysis to show the most effective measures for diagnosing CTS; therefore, future research should carry out meta-analyses to identify the most effective criteria for ultrasound, NCS, and electromyography CTS diagnosis.

## Conclusions

Ultrasound, NCS, and electromyography have been shown to be effective in the diagnosis of CTS. Our analysis shows that ultrasound has good sensitivity and is comparable to electromyography and NCS; however, it has slightly higher specificity than NCS and electromyography. Therefore, ultrasound can be used as an alternative to electrodiagnostic testing as a confirmatory test for CTS by optimizing the specificity at the expense of sensitivity. Similarly, there has been a preference for ultrasound in the diagnosis of CTS since it is cost-effective, fast in diagnosis, and painless. However, this does not mean that ultrasound can replace electrodiagnostic testing since more research needs to be done on doubtful and secondary cases of CTS. Additionally, the ability of NCS to give details on the physiological fitness of the median nerve across the carpal tunnel, grading of severity, and excluding polyneuropathy and radiculopathy make it difficult for ultrasounds to replace it. The systematic literature review has also shown that the widely used ultrasonography measurement is CSA of the median nerve. Therefore, future research work should be able to eliminate the use of measurements that are no longer valid, devise new techniques for investigation, and determine their clinical utility. Additionally, no literature has been able to correlate ultrasound measures to the severity of CTS; thus, more research should be done to investigate the correlation of these measures.
